# 疑似蛇毒液样品的纳升级超高效液相色谱-高分辨质谱分析鉴定

**DOI:** 10.3724/SP.J.1123.2022.08009

**Published:** 2023-02-08

**Authors:** Zehua LI, Chuang WANG, Bin XU, Jia CHEN, Ying ZHANG, Lei GUO, Jianwei XIE

**Affiliations:** 1.军事科学院军事医学研究院毒物药物研究所，抗毒药物与毒理学国家重点实验室，北京 100850; 1. State Key Laboratory of Toxicology and Medical Countermeasures，Institute of Pharmacology and Toxicology，Academy of Military Medical Sciences，Beijing 100850，China; 2.中央民族大学药学院，民族医药教育部重点实验室，北京 100081; 2. Key Laboratory of Ethnic Medicine，Ministry of Education，College of Pharmacy，Minzu University of China，Beijing 100081，China; 3.北京市公安司法鉴定中心，法庭毒物分析公安部重点实验室，北京 100192; 3. Forensic Science Service of Beijing Public Security Bureau，Key Laboratory of Forensic Toxicology，Ministry of Public Security，Beijing 100192，China

**Keywords:** 纳升级超高效液相色谱, 高分辨质谱, 蛇毒, 蛋白质组学, 平行反应监测, nano-ultra-high performance liquid chromatography （Nano LC）, high resolution mass spectrometry （HRMS）, snake venom, proteomics, parallel reaction monitoring

## Abstract

针对5个疑似蛇毒毒液及其沾染样品，基于纳升级超高效液相色谱-四极杆-静电场轨道阱高分辨质谱（Nano LC-MS/HRMS）技术，结合尺寸排阻色谱分离，建立了一种蛋白质种类及物种归属的严格鉴定方法。5个样品经尺寸排阻色谱分离后均得到3个洗脱峰，分别冻干后以胰蛋白酶进行溶液内酶解处理并进行液相色谱-高分辨质谱分析鉴定。首先采用全扫描-数据依赖型MS/MS（Full MS/dd MS^2^）采集模式对样品中的肽段信息进行非靶向采集，依次与Swiss-Prot、蛇亚目（Serpentes）、游蛇科（Colubroidea）、眼镜蛇科（Elapidae）、眼镜蛇亚科（Elapinae）、眼镜蛇属（*Naja*）蛋白质数据库逐级收缩比对；再筛选符合肽谱匹配度、肽段错误发现率小于1%和特征肽段数目大于等于2的蛋白质，共鉴定到32种蛋白质均来自中华眼镜蛇（*Naja atra*），可归属于*Naja atra*的10个家族，主要为三指毒素、金属蛋白酶、磷脂酶A2等。最后，采用平行反应监测模式选取每种蛋白质的两条特征肽段进行靶向验证，当两条特征肽段均满足“至少75%的y^+^和b^+^离子的Δ*m/z*小于5 ppm”时，方认为鉴定到了样品中的某一蛋白质。最终鉴定出5个样品均含有*Naja atra*蛇毒。此鉴定方法研究系统、严格，可为蛇毒中毒司法鉴定以及毒药物研究等提供有效的技术支持。

## Abstract (en)

Snake venom is a complex mixture secreted from the glands of poisonous snakes， which contains proteins， peptides， lipids， nucleosides， sugars， amino acids， amines， metal ions， and other components. According to the toxicological classification， snake venoms can be classified as neurotoxins， anticoagulants and procoagulant toxins， cardiac toxins， other toxin proteins， and enzymes. Proteins and peptides are the key components of snake venom. The establishment of rapid， accurate analysis and identification methods for proteins in snake venom is a prerequisite for snake venom-related forensic identification， intoxication events， and pharmaceutical development. Until now， the classical analysis and identification methods have mainly been biochemical or immunoassays for DNA or proteins， such as polymerase chain reaction， agglutination test， enzyme-linked immunosorbent assay， fluorescent immunoassay， and various biosensing approaches. These methods have some limitations such as a high false-positive ratio， low sensitivity， poor anti-interference ability， and limited species discrimination capability.

In recent years， with the rapid development of mass spectrometry （MS） techniques， the proteomics of snake venom has also attracted much attention and has contributed to the identification of snake species， in which non-targeted and targeted proteomics represent two main divisions. However， species identification via proteomics is in its infancy in forensic science. First， the tandem MS spectra of peptide sequences are highly complex， which poses a great challenge for the strict and accurate matching of peptides based on the rational speculation of MS fragmentation rules and theoretical calculations in non-targeted proteomics. Second， for the confirmation and identification of unknown substances， reference substances are commonly needed， but those for snake venom are lacking. Proteomics in snake venom identification is still in progress to improve the identification confidence and clarify the identification rules. In this work， a method based on nano-ultra-high performance liquid chromatography-quadrupole-orbitrap high-resolution mass spectrometry （Nano LC-MS/HRMS） and size exclusion chromatography （SEC） was developed for identifying proteins and their source species， with strict rules for five suspected snake venom samples and their contamination in one case. Three SEC elution peaks were obtained from each of the five samples， which were lyophilized and treated with trypsin in solution， and then separated and analyzed by Nano LC-MS/HRMS. First， the Full MS/dd MS^2^ mode was used for the non-targeted acquisition of peptide information in the samples， and after submission to the Swiss-Prot database， the protein databases of Serpentes， Colubroidea， Elapidae， Elapinae， and *Naja* were contracted stepwise and compared. A total of 32 proteins from *Naja atra* were identified under the conditions of both peptide spectrum match and false discovery rate less than 1%， and number of characteristic peptides greater than or equal to two. All of these were derived from ten families of *Naja atra*， mainly three-finger toxins， metalloproteinases， and phospholipase A2. Proteins D3TTC2， D5LMJ3， Q7T1K6， Q9DEQ3， and Q9YGI4 were the most common among the five samples. Finally， the parallel reaction monitoring mode was adopted to select two unique peptides for each protein for targeted verification. It was considered that a protein in the samples was truly identified when it met the strict standard “the Δ*m/z* of at least 75% y^+^ and b^+^ ions of each unique peptide was less than 5 ppm”. After these consequently procedures， we identified that all five samples contained the venom of the *Naja atra*. Our identification method is a systematic and strict example that can provide effective technical support for the forensic identification of snake venom poisoning， as well as for pharmaceutical development toward snake venoms.

世界范围内，疑似蛇毒中毒事件经常发生，其中蛇毒确证鉴定、种属来源剖析成为该类事件判定的关键。每年多达270万人因毒蛇咬伤中毒，其中8.1~13.8万人因蛇咬伤致死^[1,2]^。世界上已发现蛇类3000余种，其中毒蛇约有650种，对人体有致命威胁的毒蛇300种。我国有蛇类210种，隶属9科53属，其中毒蛇有100多种^[3]^，但剧毒蛇类仅10余种，主要为眼镜蛇、蝮蛇、竹叶青蛇、银环蛇等^[4]^。蛇毒是毒蛇从毒腺中分泌出来的复杂混合物，含有蛋白质、多肽、脂类、核苷、糖类、氨基酸、胺类、金属离子等，主要成分则为蛋白质和多肽^[5]^；按毒理学分类可分为神经毒素、抗凝及促凝血毒素、心脏毒素等各种毒素蛋白以及酶。蛋白质和肽是蛇毒发挥毒性和药性作用的关键组分。美国食品药物管理局（Food and Drug Administration， FDA）已批准源自蛇毒的抗血栓药盐酸替罗非班注射液和降压药卡托普利上市^[6]^。目前蛇毒在抗肿瘤、抗菌、抗病毒方面的作用也逐渐引起人们的重视。近日Freire等研究发现巴西矛头蝮蛇毒的衍生物可以抑制非洲绿猴肾细胞（Vero细胞）中新冠病毒的复制，抑制率可达75%^[7]^。快速、准确的蛇毒蛋白质分析鉴定方法的建立，对蛇毒中毒司法鉴定、中毒救治以及蛇毒药物的研发具有重要意义。

针对蛇毒鉴定，其经典分析方法主要为针对DNA或蛋白质的生化或免疫测定，例如聚合酶链反应、凝集测试、酶联免疫吸附检测、荧光免疫检测、生物传感等^[8]^。但均存在假阳性率高、灵敏度低、抗干扰能力差、种属区分度有限等局限性。近年来，随着质谱技术的飞速发展，蛇毒毒液蛋白质组学研究亦受到重视^[9]^。目前，蛇毒蛋白质组学研究步骤分为粗毒分离、质谱分析及生物信息学鉴定3个部分。粗毒分离的方法包括二维电泳、尺寸排阻色谱（SEC）及高效液相色谱等；质谱分析方法包括基质辅助激光解吸电离-飞行时间质谱、液相色谱-质谱（LC-MS）、电喷雾-傅里叶变换离子回旋共振质谱等^[10]^；而基于MS-Fit或Mascot搜索引擎访问相应的蛋白质网站，对所获取质谱肽段序列查询比对，则是生物信息学鉴定的主要手段。迄今，利用蛋白质组学技术已经对游蛇科、眼镜蛇科、蝰科中的58个属100多种（亚种）蛇毒进行了研究，鉴定出了10多个蛋白质家族^[11]^。根据数据采集方式，可将蛋白质组学分为非靶向蛋白质组学和靶向蛋白质组学两类。前者信息最为丰富，而后者具有更高的特异性和灵敏度。

目前法医物证鉴定时基于蛋白质组学的种属鉴定尚处于起步阶段。主要原因如下：一方面，蛋白质组学的分析鉴定本质是基于质谱断裂规律和理论计算的合理推测，但多肽序列的二级质谱谱图具有高度复杂性，法医蛋白质组学中基于MS/MS鉴定肽段时主要使用非靶向采集后数据库匹配的途径，具有一定的错误率。中毒样品还可能存在中毒机体自身种属蛋白质的极大干扰；另一方面，针对未知物质的确证鉴定，通常采用与标准品（参考品）进行比较的方式^[12,13]^，但目前蛇毒的参考品仍缺乏。

本研究采用液相色谱-高分辨质谱技术，基于纳升级超高效液相色谱-四极杆-静电场轨道阱高分辨质谱（Nano LC-MS/HRMS）系统，针对5个疑似蛇毒样品，经胰蛋白酶溶液内酶解后，首先以非靶向模式采集数据后进行蛋白质数据库比对，获得未知样品中蛋白质的物种来源信息；然后通过控制肽谱匹配度（PSM）、肽段错误发现率（FDR）值和特征肽段的数量来提高蛋白质鉴定的准确度；再采用靶向采集模式对推断的蛋白质进行验证，最终成功鉴定出5个疑似蛇毒样品均含有来源于中华眼镜蛇（*Naja atra*）的蛇毒。该分析策略简便、严格、可靠性强，可为蛇毒中毒的司法案件鉴定、临床中毒救治以及蛇毒药物研发提供有效的技术支持。

## 1 实验部分

### 1.1 仪器与试剂、材料

EASY-nLC 1200纳升液相色谱、Orbitrap Exploris 480质谱仪购自美国Thermo Scientific公司，AKTA Pure 25色谱系统购自美国Cytiva公司，十万分之一天平R200 D购自德国Sartorious公司。Protease^MAX^表面活性剂购自美国Promega公司，胰蛋白酶（测序级）、乙腈（色谱纯）、二硫苏糖醇、碘乙酰胺均购自美国Thermo Scientific公司，低相对分子质量标准蛋白质（包含抑肽酶6.5 kDa、核糖核酸酶A 13.7 kDa、碳酸酐酶29 kDa、卵清蛋白43 kDa、伴清蛋白75 kDa 5种蛋白质）购自美国Citiva公司，三氟乙酸、甲酸（分析纯）购自北京百灵威科技有限公司，碳酸氢铵（分析纯）购自国药集团化学试剂有限公司，实验用水为Milli-Q A10型超纯水系统（美国Millipore公司）制备的超纯水（电阻率为18. 2 MΩ·cm）。5个疑似蛇毒样品均为同一种蛇毒液污染样品，来自北京市公安局司法鉴定中心（见表1）。

**表1:** 疑似蛇毒样品的性状

No.	Sample name	Character
A	powder inside newspaper	yellowish-brown
B	needles wrapped in paper	rufous
C	powder inside plastic bag	yellowish-brown
D	plastic bag	yellowish-brown
E	wine glass	contained thick yellowish liquid
		and several needles

### 1.2 实验条件

#### 1.2.1 Nano LC-MS/HRMS条件

脱盐富集柱：Thermo Acclaim PepMap^TM^100柱（2 cm×75 μm， 3 μm）；分离柱：Thermo Acclaim PepMap^TM^ RSLC纳升分析柱（25 cm×75 μm， 2 μm）。流动相A为0.1%（v/v）甲酸水溶液，流动相B为0.1%甲酸水溶液-乙腈（1∶4， v/v）；洗脱程序：0~5 min， 4%B~10%B； 5~63 min， 10%B~30%B； 63~72 min， 30%B~40%B； 72~80 min， 40%B~100%B。流速为300 nL/min；进样量为1 μL。

离子源温度：320 ℃；毛细管电压：3.6 kV；辅助气温度：320 ℃；鞘气流速：10 L/h；辅助气流速：30 L/h；采集模式：全扫描-数据依赖型MS/MS（Full MS/dd MS^2^）和平行反应监测（PRM）；碰撞能量（CE）： 15、27.5、40 eV；采集范围：200~2200 Da；电离模式：ESI正离子模式；一级质谱分辨率为35000 FWHM，二级质谱分辨率为17500 FWHM。

#### 1.2.2 Proteome Discover参数设置

搜索引擎：Sequest^HT^；数据库：Swiss-Prot全库、蛇亚目（Serpentes）、游蛇科（Colubroidea）、眼镜蛇科（Elapidae）、眼镜蛇亚科（Elapinae）、眼镜蛇属（*Naja*）蛋白质数据库；胰蛋白酶（全切）；最小肽段长度：6个氨基酸；最大肽段长度：144个氨基酸；最大漏切位点：2；碎片离子的质量误差：0.02 Da；肽段的质量误差：5×10^-6^（5 ppm）； PSM和肽段置信度均为High，肽段的FDR设为小于0.01。

### 1.3 实验步骤

#### 1.3.1 样品前处理

5个样品，每个约含0.8~1 mg蛋白质，分别加入2 mL 10 mmol/L磷酸盐缓冲液（PBS），振摇1 min后14000 g离心取上清液冻干备用。

#### 1.3.2 尺寸排阻色谱

采用Superdex 200 increase 10/300 GL预装柱（Cytiva公司）进行分子尺寸排阻色谱分离。对预装柱进行平衡后，利用10 mmol/L PBS以0.5 mL/min的流速对5个样品冻干粉的PBS溶液进行洗脱，收集洗脱得到的各个组分，冻干备用，蛋白质含量由280 nm处的峰高度估算得到（50 mAU=0.2 mg）。

#### 1.3.3 溶液胰蛋白酶酶切

取约100 μg样品，加入2 μL 1% Protease^MAX^表面活性剂后，加入250 mmol/L 碳酸氢铵使其终浓度为50 mmol/L。加入1 μL 0.5 mol/L二硫苏糖醇56 ℃孵育20 min，冷却至室温后加入2.7 μL 0.55 mol/L碘乙酰胺，室温避光孵育15 min。向上述孵育体系中加入1 μL 1% Protease^MAX^表面活性剂和1.8 μL胰蛋白酶（样品与胰蛋白酶的质量比为1∶50），于37 ℃孵育3 h。加入三氟甲酸至终浓度为0.1%（v/v），室温孵育5 min后终止反应。加入4倍体积的甲醇-乙腈（1∶3， v/v）混合液涡旋1 min后，14000 g离心取上清液浓缩至干，以0.1%甲酸水溶液复溶至50 μL。

#### 1.3.4 Nano LC-MS/HRMS分析鉴定

样品经溶液内酶解处理后，在Nano LC-MS/HRMS的Full MS/dd MS^2^模式下进行数据采集。将采集的数据信息导入Proteome Discover软件（ver. 2.5），利用Sequest^HT^搜索引擎在Swiss-Prot蛋白质数据库中搜寻匹配相关蛋白质，给出的信息包括匹配的蛋白质和肽段、肽段覆盖率、Sequest^HT^评分以及与数据库相匹配的y^+^、b^+^离子等信息^[14]^。

## 2 结果与讨论

### 2.1 质谱分析前的分离分析

进行质谱分析前，对样品进行适当的分离纯化是一种获取高蛋白质组覆盖范围、提高蛋白质鉴定数量的有效方式^[15]^。蛇毒的组成丰富、多样，且由于新鲜的蛇毒含有65%~80%的水分，新鲜蛇毒样品并不十分稳定，多数新鲜蛇毒室温下容易发生降解变质^[6]^。在目前常见的蛇毒分离途径^[8]^中，SEC简捷、快速、载样量高，一般在低温下进行分离，有利于保持样品的稳定性^[16]^。我们即采用SEC对5个样品进行了分离。如图1所示，样品均可分离得到3个谱峰且对应的洗脱体积基本一致。与低相对分子质量的标准蛋白质比较，5个样品中的蛋白质质量范围均小于43 kDa，多数蛋白质分布于6.5~29 kDa范围内，少量蛋白质小于6.5 kDa，这与蛇毒中大量的蛋白质为低相对分子质量蛋白质这一特征相符合^[17]^。

**图1:**
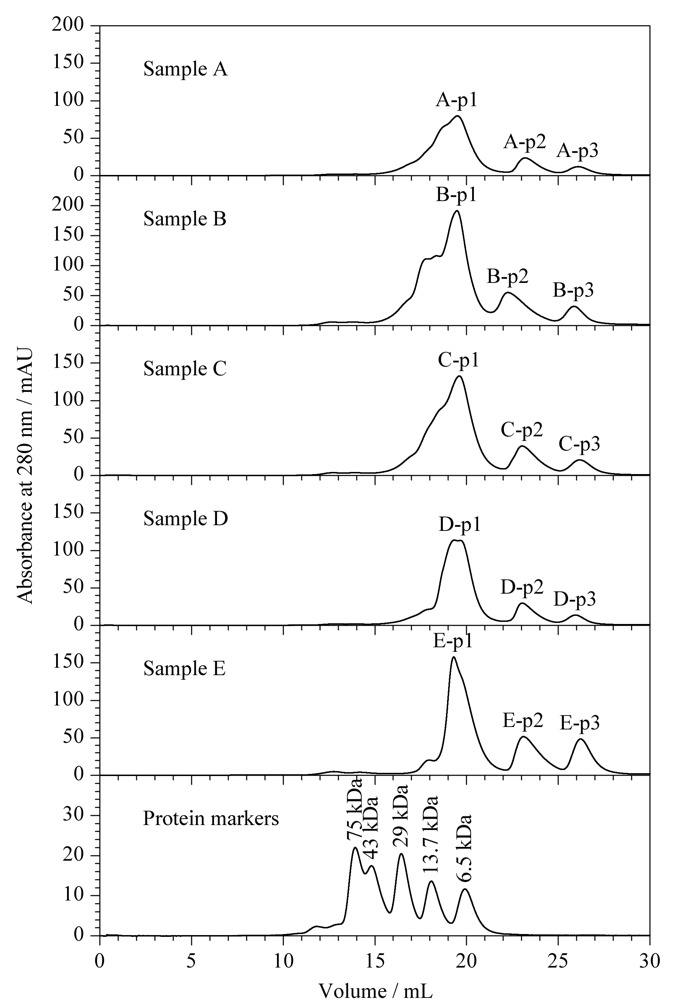
5个样品以10 mmol/L PBS洗脱的SEC色谱图

近年来Nano LC在蛋白质组学领域成为主要的分离手段，其流速为几十到几百nL/min，色谱柱单位截面积的进样浓度大于常规色谱柱，样品的稀释效应低，展现出更高的灵敏度^[18]^。因此，本研究采用Nano LC代替了UPLC用于质谱前酶解肽段的分离，针对样品E，考察了60、90、120 min 3个梯度，与Swiss-Prot数据库比对后分别可匹配到82、87和83种蛋白质，我们在此选择90 min这一梯度，为法医蛋白质组学中未知样品的鉴定提供了更丰富的信息。

### 2.2 非靶向蛋白质组学鉴定

法医蛋白质组学中未知样本的鉴定最具挑战。经数据库搜索匹配来识别多肽进行蛋白质鉴定时，数据库的选择严重影响检测到的蛋白质数量和种类。在生物医学蛋白质组学研究中常用的解决方案是将蛋白质组学与基因组学相结合，基因组学可提供样本匹配的蛋白质序列数据库用于数据库搜索匹配^[19,20]^。但当未联合测定基因组时，尽可能获取丰富信息的蛋白质组数据就成为先决条件。

我们首先采用了Full MS/dd MS^2^非靶向数据采集模式，获得样品中肽段的精确相对分子质量以及部分碎片信息，随后将其导入Proteome Discover软件，利用Sequest^HT^搜索引擎进行蛋白质数据库匹配。对于来自5个样品的15个SEC组分，分别进行多次蛋白质数据库搜索匹配，每次根据上一次的搜库结果不断缩小数据库规模，以提高推断未知样品蛋白质物种来源的准确度。大型数据库的搜索匹配可提供样本最丰富的信息，但过多与样品不相关的蛋白质数据会增加假阳性率，而本研究中采用的“减小数据库规模将多肽分配到适当的分类级别”这一途径可解决这一问题。

另外，从PSM、肽段置信度和特征肽段数目方面，进一步提高未知样品中蛋白质物种来源的推断准确度。国际人类蛋白质组组织^[21]^和国际禁止化学武器组织^[22]^提出的指南中，分别明确强调了人类蛋白质和蓖麻毒素法医检测时特征肽段的重要性，且均规定至少需要两条特征肽段来进行蛋白质鉴定。因此，参考上述指南，我们将PSM和肽段的FDR值控制在1%以下，特征肽段的数目≥2，符合目前蛋白质鉴定中的较严格标准。

首先搜索匹配了经验证和注释的涵盖所有物种蛋白质的Swiss-Prot数据库，去除污染物库后所有特征肽段数目≥2的蛋白质其物种来源均为蛇，15个组分得到的蛋白质总数为84。将数据再次提交至涵盖所有与蛇相关蛋白质的蛇亚目数据库，所有特征肽段数目≥2的蛋白质其物种来源均为游蛇科，得到的蛋白质总数为117。再次将数据提交至涵盖所有游蛇科相关蛋白质的游蛇科数据库，发现所有特征肽段数目≥2的蛋白质其物种来源均为眼镜蛇科，得到的蛋白质总数为114。

如图2所示，搜索Elapidae数据库可匹配到Acanthophiinae、Bungarinae、Elapinae、Laticaudinae、Notechinae 5个眼镜蛇亚科的蛋白质125种，其中Elapinae亚科的蛋白质数量为107种，占鉴定蛋白质总数的85%以上。因此，我们将数据库缩小至涵盖所有眼镜蛇亚科相关蛋白质的Elapinae数据库，15个组分可匹配到*Naja*、*Ophiophagus*、*Dendroaspis polylepis*、*Hemachatus*、*Walterinnesia*、*Micrurus* 6个属的蛋白质共113种，其中*Naja*属的蛋白质数量为100种，占鉴定蛋白质总数的88%以上。最后，将数据库锁定至涵盖所有眼镜蛇属相关蛋白质的*Naja*数据库，其中特征肽段数目≥2的蛋白质其物种来源可归属至*Naja atra*、*Naja kaouthia*、*Naja naja*、*Naja pallida*、*Naja mossambica*、*Naja sagittifera*、*Naja sputatrix*、*Naja melanoleuca*、*Naja oxiana*、*Naja annulifera*、*Naja haje haje*、*Naja samarensis* 12个种，其中物种来源归属为*Naja atra*的蛋白质数目最多，有32种。因此推断样品含有来自*Naja atra*的蛇毒。

**图2:**
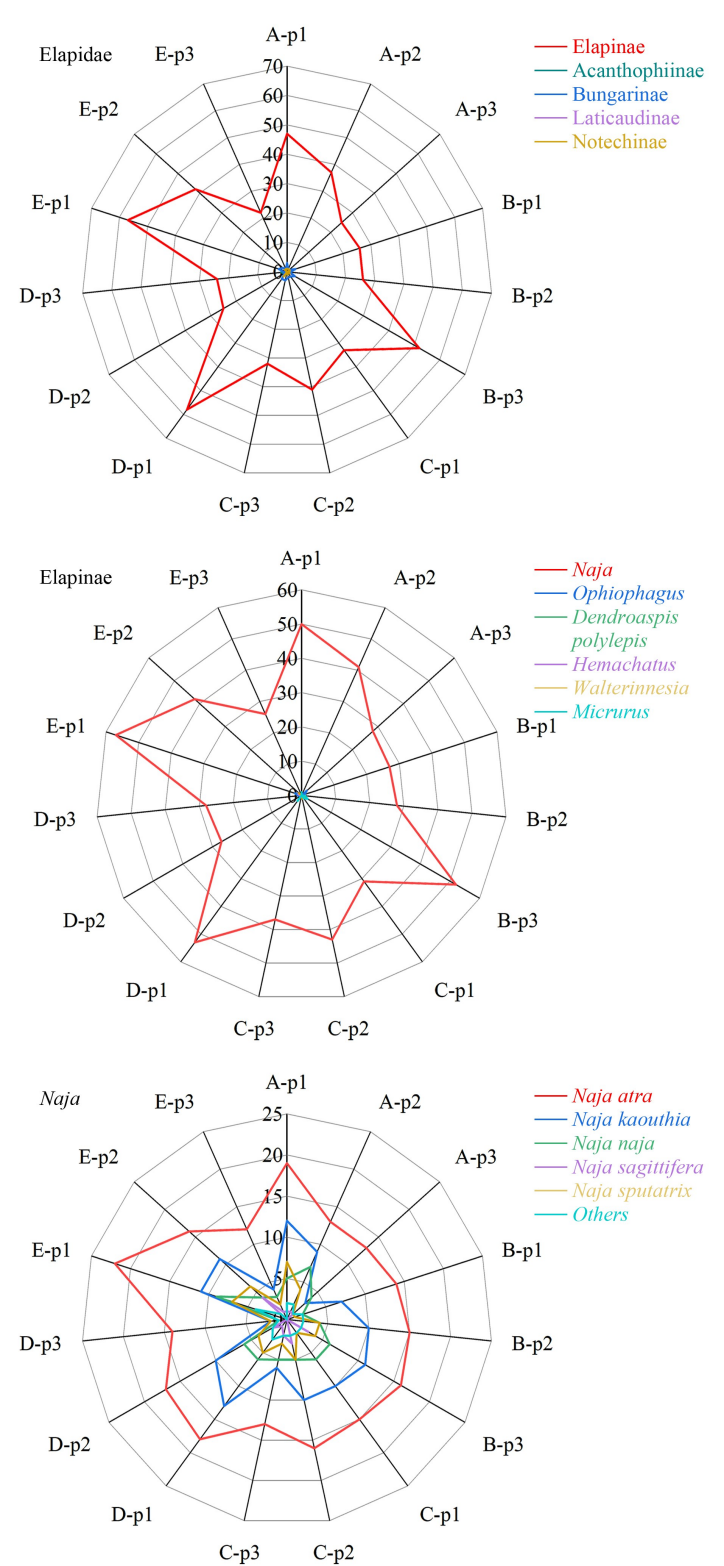
5个样品的蛋白质数据库匹配结果

从以上逐级收缩匹配到的蛋白质数量可以看出，蛇物种数据库匹配到的蛋白质数量反而小于蛇亚目、游蛇科、蛇亚科等数据库，原因在于当设定同样的FDR阈值范围（<1%）时，数据库越大，诱饵库也越大，导致符合FDR阈值的PSM和肽段数目降低，鉴定到的蛋白质数目可能相应减少。

对鉴定到的蛋白质进行比较（见图3）， 5个样品的SEC第1、2、3个洗脱峰分别鉴定到来自*Naja atra*的26、26、22种蛋白质，其中10、5、5种蛋白质为共有蛋白质；5个样品的3个SEC洗脱峰共鉴定到32种蛋白质，其中D3TTC2、D5LMJ3、Q7T1K6、Q9DEQ3、Q9YGI4为共同鉴定到的蛋白质。图4列举了其经非靶向蛋白质组学鉴定到的蛋白质种类及其Sequest得分。

**图3:**
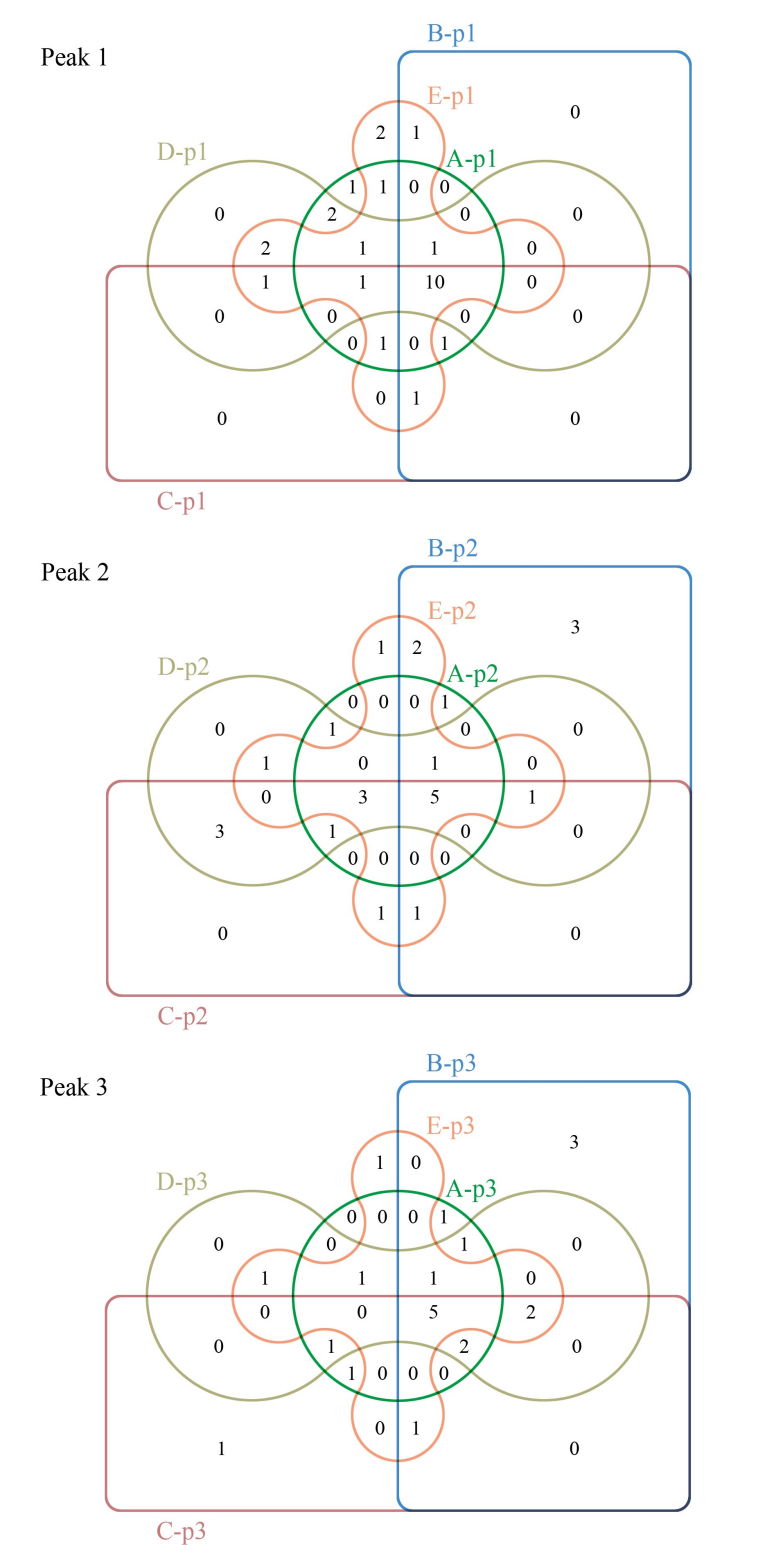
3个SEC洗脱峰鉴定到的来自*Naja* atra的蛋白质

**图4:**
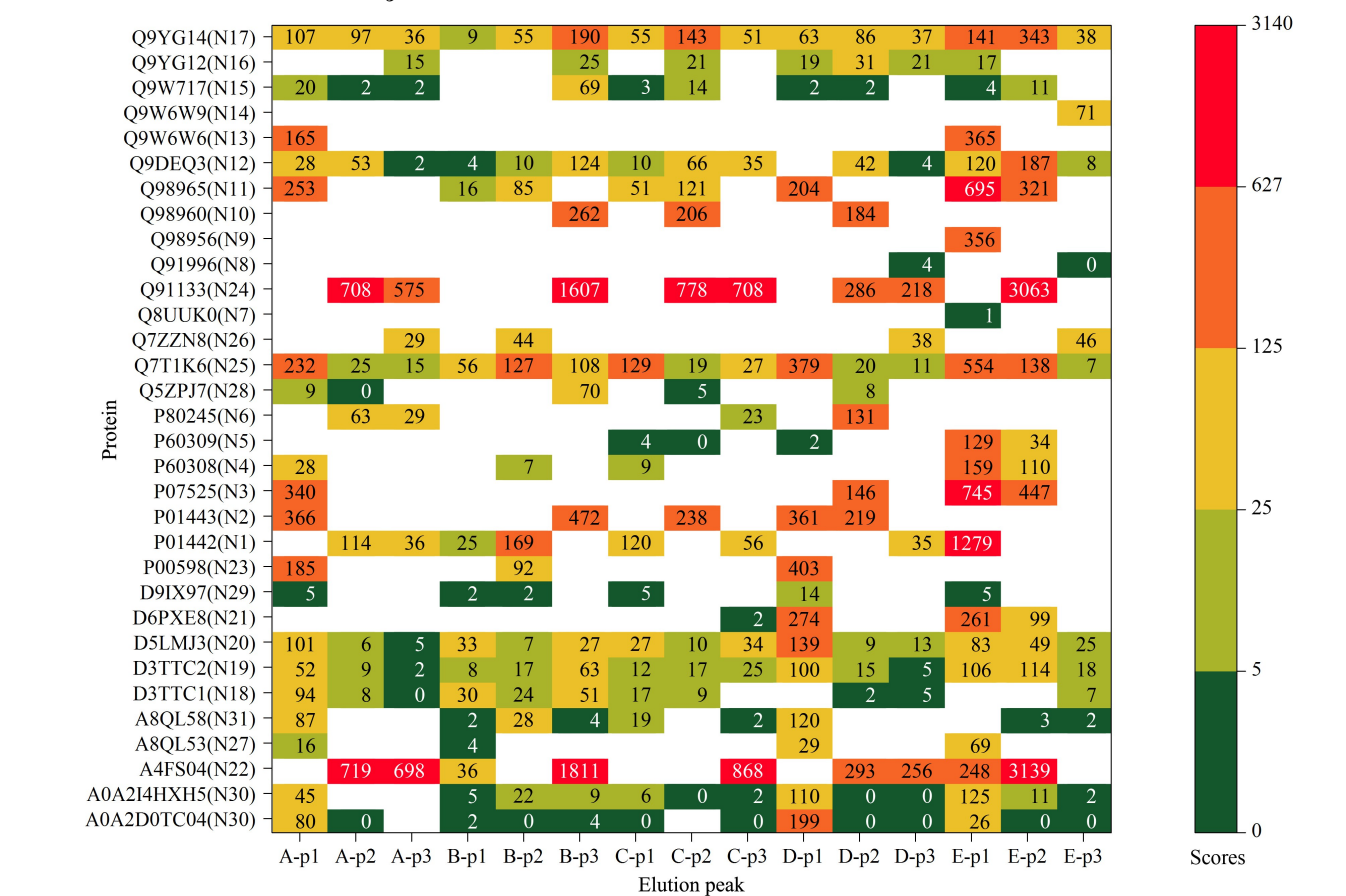
5个样品的SEC洗脱峰鉴定到的蛋白质

### 2.3 靶向蛋白质组学鉴定

在法医蛋白质组学领域，进行未知样品的靶向蛋白质组学研究需要通过非靶向蛋白质组学提供样品所含的蛋白质信息或对应的物种信息。这种非靶向筛查与靶向验证相结合的分析策略可提高未知样品中蛋白质鉴定的准确度^[14]^。在确认样品的蛋白质或者物种来源信息后，常选取与目标蛋白质的特征肽段保留时间和MS/MS信息比对的策略进行样品中蛋白质的分析鉴定。

考虑到多肽的MS/MS谱图复杂性高，且相近物种存在多种同源性多肽的现象，一条多肽特征肽段并不足以归属到特征蛋白质。在此，我们对非靶向筛查鉴定到的32种来自*Naja atra*的蛋白质采用每种蛋白质选取两条特征肽段的PRM模式进行靶向验证。国际禁化武组织指南认为^[22]^，当两条特征肽段均满足至少75%的与理论碎片相匹配的y^+^和b^+^离子的Δ*m/z*小于5 ppm时，可确证相应蛋白质的存在。我们即在此采用该严格标准以尽可能排除假阳性。图5展示了进行PRM靶向验证的蛋白质的提取离子流色谱图。每个样品中的3个SEC组分在非靶向筛查中鉴定到的全部蛋白质均符合该标准。因此，进一步确证出5个样品中蛋白质的物种来源均为*Naja atra*。

**图5:**
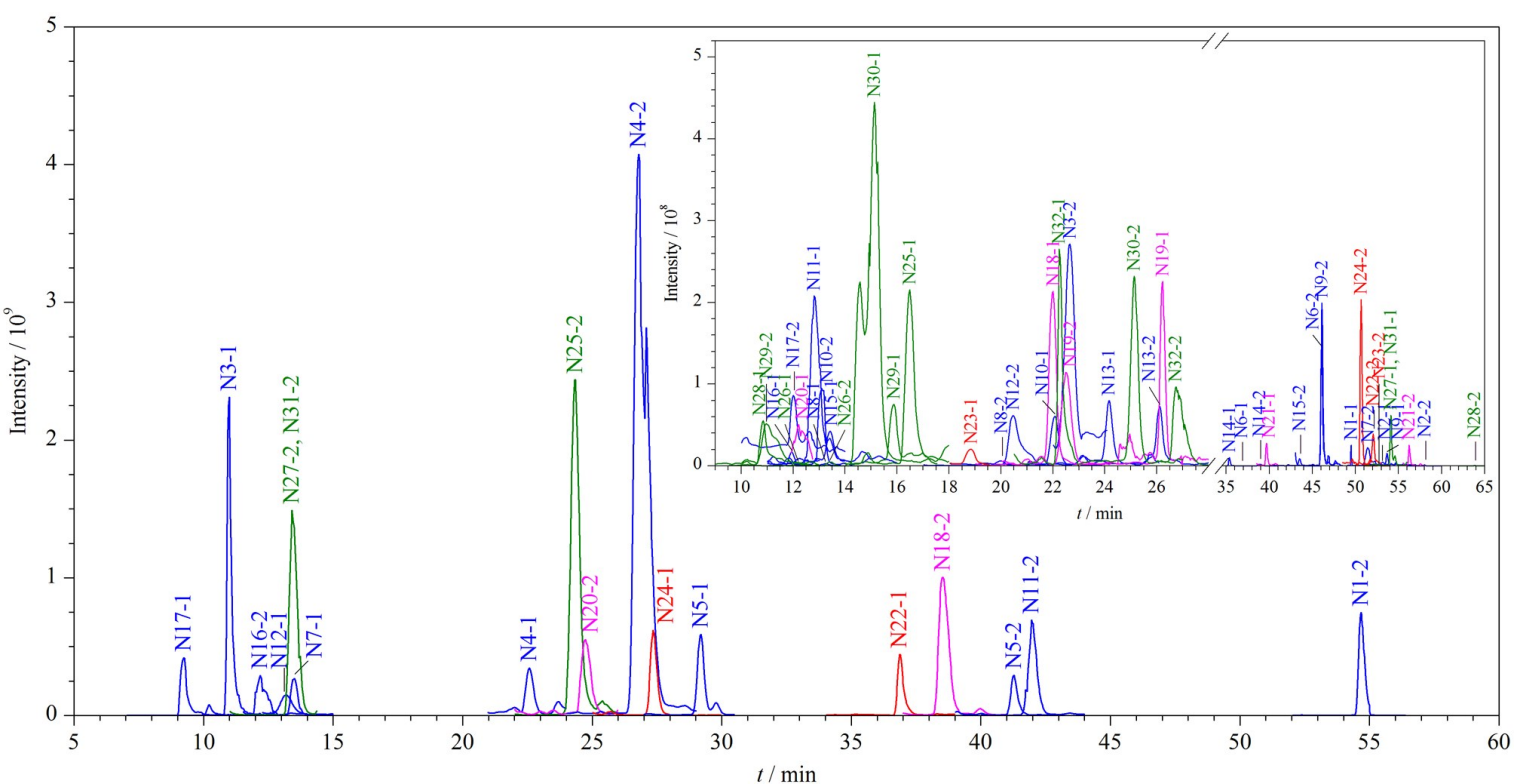
PRM验证蛋白质的特征肽段提取离子色谱图

在靶向验证中，特征肽段的界定与选择以及使用两条特征肽段对蛋白质进行确证是否足够充分或过于严格等问题值得后续探究。目前，这种方法可能存在两方面的问题，一是低估非特征肽段所提供的信息，二是肽段的特征性与选取的蛋白质数据库相关。当只考虑人类蛋白质组时，一种特定人类蛋白质的特征肽段可能在哺乳动物中广泛共享^[23]^。此外，随着测序技术的发展，蛋白质序列数据库将不断扩大，新增加的蛋白质亦会造成原有特征肽段可能失去其特征性^[13]^。

我们对经靶向验证的32种蛋白质进行了进一步的分类分析，确定其可归属于*Naja atra*的10种蛋白质家族，图6展示了它们之间的进化关系。这10种蛋白质家族分别是三指毒素（17种）、金属蛋白酶（4种）、磷脂酶A2（3种）、富含半胱氨酸的分泌蛋白（2种）、肽酶S1（1种）、kunitz肽（1种）、利钠肽（1种）、5'-核酸酶（1种）、黄素单胺氧化酶家族（1种）和核苷酸焦磷酸酶（1种）。三指毒素是眼镜蛇毒液中含量最丰富的蛋白质家族^[24]^，可大致分为细胞毒素、*α*-神经毒素、*κ*-神经毒素和毒蕈碱毒素^[25]^。我们共鉴定到12种细胞毒素，这与眼镜蛇毒液又被归类为细胞毒性毒液^[26]^相符合。另一方面，较之我们以前的针对样品E的分析鉴定工作^[14]^，该工作使用UPLC-MS/MS，使用Swiss-Prot注释验证库和Uniprot注释验证和未验证库，鉴定到归属于*Naja atra*、*Naja naja*、*Naja kaouthia*、*Naja melanoleuca*、*Naja sputatrix*、*Naja nivea*、*Naja annulifera*的共计15种蛋白质，此次鉴定到的32种来自*Naja atra*的蛋白质涵盖了原鉴定到的10种*Naja atra*蛋白质中的7种，但多鉴定出25种蛋白质，此点归属于Nano LC提升灵敏度以及使用较长梯度洗脱程序（90 min）的贡献。未鉴定到的3种蛋白质中，P60304和P80958在本工作中仅归属了一条特征肽段，未匹配到E2IU03。

**图6:**
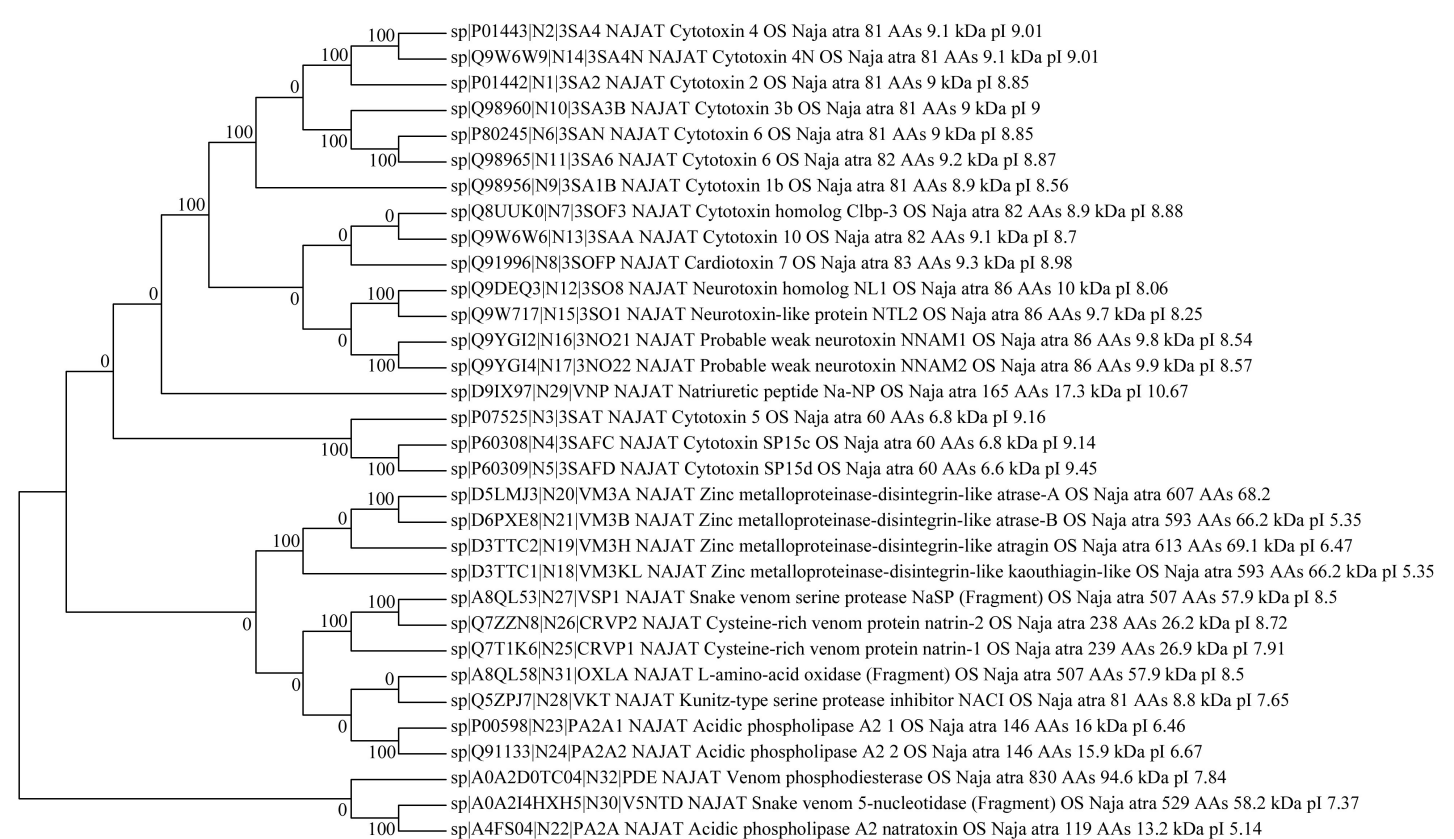
来自于5个样品的32种鉴定蛋白质的进化树

## 3 结论

本研究采用Nano LC-MS/HRMS法分析鉴定疑似蛇毒样品中蛋白质的物种来源。首先对经尺寸排阻色谱分离和胰蛋白酶溶液内酶解的样品进行非靶向采集，提出多次收敛蛋白质数据库比对的思路，在5个样品的3个SEC洗脱峰中共鉴定到32种来自*Naja atra*的蛋白质。蛋白质数据库的逐级收敛结合严格肽段鉴定规则（PSM、肽段FDR值、特征肽段数量）有效提高了蛋白质的鉴定准确度。然后，采用PRM靶向采集模式结合肽段理论碎片严格阈值的手段对样品中鉴定到的蛋白质进行验证。最终成功鉴定出5个疑似蛇毒样品均含有来源于*Naja atra*的蛇毒。该分析策略可为蛇毒中毒的司法案件鉴定、临床中毒救治以及蛇毒药物研发提供有效的技术支持。
